# Thermal and Mechanical Properties of Expanded Graphite/Paraffin Gypsum-Based Composite Material Reinforced by Carbon Fiber

**DOI:** 10.3390/ma11112205

**Published:** 2018-11-07

**Authors:** Bo Zhang, Yuanyuan Tian, Xiaoyan Jin, Tommy Y. Lo, Hongzhi Cui

**Affiliations:** 1 Guangdong Provincial Laboratory of Durability for Marine Civil Engineering, Shenzhen University, Shenzhen 518060, China; 2172332351@email.szu.edu.cn (B.Z.); 2150150417@email.szu.edu.cn (Y.T.); 2172332306@email.szu.edu.cn (X.J.); 2 Department of Architectural and Civil Engineering, City University of Hong Kong, Hong Kong 518000, China; lo.tommy@cityu.edu.hk

**Keywords:** phase change material, expanded graphite, thermal energy storage, gypsum-based composite material, carbon fiber, mechanical properties, thermal performances

## Abstract

Phase change material (PCM) is a kind of thermal energy storage material. Solid-liquid PCM composite materials must overcome the issues of material leakage and low thermal conductivity before they are suitable for widespread use in the fields of building and industry. In this study, porous expanded graphite (EG) is used as a carrier, which absorbs the PCM to fabricate EG/paraffin composites (EG/P) containing 90.6% paraffin, and a latent heat of up to 105.3 J/g was measured. Because gypsum board is widely used in buildings, therefore, EG/P composites are suitable to be integrated into gypsum to develop expanded graphite/paraffin gypsum-based composite material (EGPG) for thermal energy storage. In order to optimize the performance of EGPG, carbon fiber (CF) is used to reinforce their thermal and mechanical properties. The test results show that when 1 wt % CF is incorporated into the EGPG, the thermal conductivity increased 36.0%, and thus EGPG shows superior thermal control through the significantly increased efficiency of heat transfer. After 1 wt % CF was added, the flexural and compressive strength of EGPG were increased by 65.6% and 6.4%, respectively. The improved thermal and mechanical performance of EGPG modified by CF demonstrates that it is a structural-functional integrated building material suitable for building envelope system.

## 1. Introduction

Energy consumption in the construction industry accounts for 40% of the total energy consumption and 30% of the annual greenhouse gas emissions [1]. Global energy reserves are becoming increasingly overwhelmed, thus energy-saving and environmentally friendly materials must be used to reduce the consumption of energy. This has motivated research into the development of thermal energy storage solutions, including sensible heat storage, latent heat storage, and electrochemical energy storage [2,3,4]. Latent heat storage is widely used in various fields, such as solar thermal energy storage and industrial waste heat recovery, due to its high energy storage density and isothermal phase transition temperature [5,6]. Additionally, the use of air-conditioners has increased as the thermal comfort index increases to meet the demand of higher living standards. Therefore, the use of latent heat storage materials, such as phase change materials (PCMs), combined with air conditioning systems in buildings may be used to significantly reduce energy consumption.

PCMs include both organic and inorganic PCMs. So far, inorganic PCMs are restricted in practical applications due to supercooling and phase segregation [7]. Organic PCMs are chemically inert and have good stability and corrosion resistance [8,9]. Paraffin, a typical organic phase change material, has been widely used because of its many advantages, including non-toxicity, stable chemical properties, no phase separation, low volume change during the phase change process, high enthalpy, and low cost [10]. However, the low thermal conductivity (about 0.24 W/m·K) of paraffin limits its widespread application because of inefficient heat transfer when absorbing or releasing thermal energy. In order to improve the thermal performance of paraffin as a PCM, its low conductivity should be modified.

Carbon-based materials are widely used for their high thermal conductivity or excellent mechanical properties. To improve the thermal management of insulated gate bipolar transistors (IGBT), Chang et al. [11] investigated the effects of nano-graphite sheet loading on paraffin to the thermal conductivity and shape-stability. They reported that the thermal conductivity of paraffin mixed with 15 wt % nano-graphite sheets was improved by five times compared to that of pure paraffin, and paraffin/nano-graphite sheets demonstrated greater shape stabilized properties. In addition, expanded graphite (EG) has been recognized for its low density, high porosity, high thermal conductivity, low price, and compatibility with organic phase change materials [12,13]. Hence, incorporating EG into paraffin could significantly increase its thermal conductivity and improve thermal transfer efficiency. Also, porous EG could be used as a carrier to absorb PCM to form a stabilized-shape phase change material (SSPCM), which could then prevent leakage of the paraffin during the process of melting. According to the research of Yang et al. [14], EG could absorb about 93.1% paraffin and the latent heat of the EG/paraffin was up to 152.8 J/g, which has potential for thermal energy storage applications. However, a cement paste containing 10 wt % EG/paraffin was found to have a compressive strength of 14.6 MPa, a reduction in compressive strength of 77.9% compared with pure cement paste. In practice, structural-functional integrated building materials are not only required to meet energy storage performance, but also must have a higher mechanical performance.

Carbon fiber has been used to reinforce composite materials due to its high tensile strength and thermal conductivity [15,16]. For example, Han et al. [17] proposed that after mixing 0.8 wt % CF into cement, the flexural strength is increased by 14.6% relative to a control sample without fiber. They explained that CF with high tensile strength could restrain the growth of cracks and it bonds well with hydration products, and thus it could enhance the flexural strength of cement by absorbing energy. However, they also mentioned that the addition of carbon fiber would cause a decrease of compressive strength due to the generation of a larger air void inside the matrix. Similar to Han et al. [17], Cui et al. [18] also found that CF helped to retrain crack pulling and thus improved the mechanical strength of cement. They presented, in that study, that the flexural strength of cement paste and alkali-activated slag mixed with 1 wt % CF was increased by 130.8% and 302.6%, respectively, after 28 days, compared with a control. The above research both illustrate that CF can be used to solve the matter of thermal energy storage building materials with low strengths. Apart from the reinforcement of mechanical strengths, Jiang et al. [19] used carbon fiber to prepare carbon bonded carbon fiber monoliths/PCM, and found that the in-plane thermal conductivity of the PCM composites was improved by 57 times over pure PCM, while the out-of-plane thermal conductivity was increased by 5.5 times. In addition, they showed that there was a linear relationship between the volume fractions of CF in the PCM composite with the improvement of thermal conductivity. Further improvement of thermal conductivity could achieve a higher heat transfer efficiency of PCM. In a word, CF is a promising candidate for enhancing the thermal and mechanical properties of structural-functional integrated building material.

Gypsum board usually was used as interior walls and ceiling board, while it does not have an energy storage function. If PCM was successfully integrated with the gypsum matrix, a structural-functional integrated gypsum board might be obtained and its application in building can be very wide-ranging [20,21]. Therefore, in this study, EG is used as a carrier to contain paraffin to fabricate EG/P with the aim of minimizing leakage and improving the thermal conductivity of paraffin. Besides, the morphology, thermal performance, and chemical structure of EG/P are characterized by scanning electron microscopy (SEM), differential scanning calorimeter (DSC), thermogravimetric analysis (TGA), and Fourier transform infrared (FT-IR) spectroscopy accordingly before it is incorporated into the gypsum matrix. Finally, the thermo-regulated performance and mechanical strengths of EGPG with or without CF was tested by means of a self-designed room model testing system.

## 2. Experimental

### 2.1. Materials

**Figure 1:**
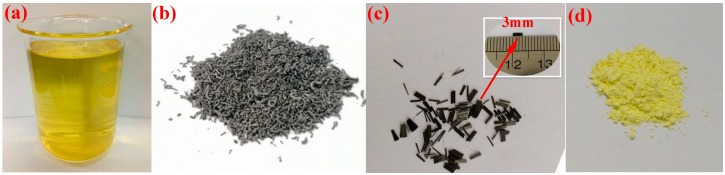
The morphology of the raw materials: (**a**) Paraffin; (**b**) EG (Expanded Graphite); (**c**) carbon fiber; (**d**) gypsum.

The paraffin used in the experiment, shown in Figure 1a, was purchased from Sinopec Group and has a latent heat of approximately 116.2 J/g. The melting and freezing temperatures are 20.8 °C and 19.4 °C, respectively, which is within the human comfort level (16–26 °C) [22]. The expanded graphite (EG), shown in Figure 1b, was supplied by Teng-sheng-da Carbon Machinery Co., Ltd. (Qingdao, China), and has an expansion ratio of 300. Polyacrylonitrile-based short carbon fibers (PAN-CF, 700SC-12K), shown in Figure 1c, with an average diameter of 7 μm and length of 3 mm, were purchased from Guangzhou Kaben Composite Materials Co., Ltd. (Guangzhou, China). The gypsum powder, shown in Figure 1d, was super hard gypsum with an initial setting time of 8–10 min and was purchased from Guangzhou Bosheng Gypsum Products Co., Ltd. (Guangzhou, China).

### 2.2. The Preparation and Characterization of EG/P

**Figure 2:**
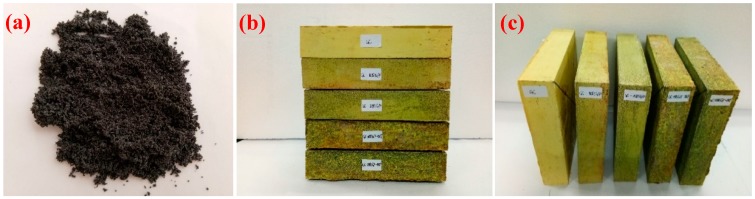
The morphology of the prepared materials: (**a**) Appearance of the prepared EG/P; (**b**,**c**) prepared gypsum board.

In this experiment, EG is used as a carrier for the paraffin. Firstly, the vacuum impregnation method [23] was used to press liquid paraffin into the mesopores of EG, then the mixture of EG/paraffin (EG/P) composite and liquid paraffin was filtered to remove excess paraffin. After filtering the solution, EG/P was drained on high adsorptive paper, and the paper placed in the oven at 60 °C. The paper was replaced frequently until the excess paraffin was removed and EG/P became a constant mass. Then, the EG/P (as shown in Figure 2a) could be collected.

To investigate fully the performance of the EG/P, many tests were conducted. DSC (Differential Scanning Calorimeter, DSC-200F3, Selb, Germany) was used to measure the melting and freezing temperature and enthalpy of the paraffin and EG/P. The temperature range of the measurement process was 0 °C to 60 °C, with a heating/cooling rate of 1 °C/min. A continuous nitrogen flow passes though the measurement system at a rate of 50 mL/min. The thermal stability of EG/P was determined by TGA (Thermal Analysis System, TGA Q50, New Castle, DE, USA). The sample was tested in a nitrogen atmosphere in a temperature range of 0–600 °C, using a heating rate of 10 °C/min. The chemical structure of paraffin and EG/P was detected by FT-IR (Fourier Transform Infrared Spectrometer, Perkin Elmer Spectrum 100, Toronto, ON, Canada), and the test wavelength ranged from 500 to 4000 cm^−1^. The microstructure of EG and EG/P was observed by FESEM (Field emission scanning electron microscope, SU-70, Tokyo, Japan) in the secondary electron mode.

### 2.3. The Preparation and Characterization of EGPG

#### 2.3.1. The Sample Preparation of EGPG

The mix proportion of EGPG is shown in Table 1. The procedures of sample preparation are as follows: Firstly, gypsum powder and EG/P were mixed and stirred for two minutes to ensure even dispersion of EG/P before water and CF were added into the stirring pot. The mixture was then stirred for 3 min at low-speed and a further 1 min at high-speed. After mixing, the EGPG was poured into one of three steel molds measuring either 40 mm × 40 mm × 160 mm to test the mechanical strength, 40 mm × 200 mm × 200 mm to test the thermos-regulated performance (shown in Figure 2b,c), or Φ30 mm × 40 mm to test the thermal conductivity. After 1 h, the samples were removed from the mold and then were kept indoors in a temperature and humidity-controlled environment (20 ± 1 °C and 60% RH) for 7 days before testing.

#### 2.3.2. Thermal Conductivity Test

Thermal conductivity is one of the most important parameters for any PCM because it determines the heat transfer efficiency of the PCM. The thermal conductivity of EGPG were investigated using a thermal conductivity tester (XIATECH, TC3000, Xian, China) based on the hot wire method [24]. Each reported value was the average of three samples, and the maximum standard deviation measured was less than 0.002 W/(m·K). The measurement temperature ranged from 19 °C to 21 °C.

#### 2.3.3. Thermo-Regulated Performance Test

**Figure 3:**
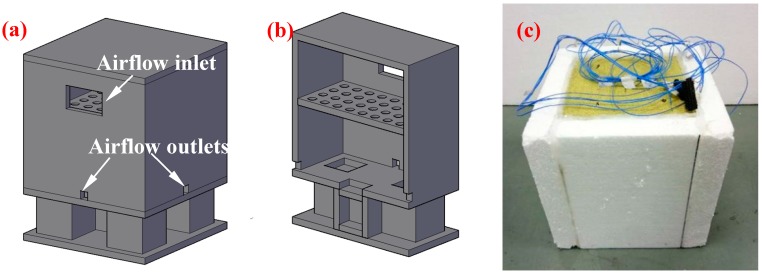
Schematic diagrams of thermal performance testing: (**a**) Testing system; (**b**) section of the testing system; (**c**) small house model.

The thermal-regulated performance of EGPG was measured through a self-designed room model [25], as shown in Figure 3a. A foam environmental chamber, as shown in Figure 3b, with internal dimensions of 1000 mm × 1000 mm × 1000 mm, was made to create a uniform temperature environment surrounding the small test room during heating and cooling. A small test room 290 mm × 290 mm × 285 mm in size was built. The test panel of EGPG with a size of 40 mm × 200 mm × 200 mm was placed on the top, acting as a ceiling panel. All sidewalls were made of polystyrene foam sheeting with a thickness of 45 mm. The internal dimension of the room was 200 mm × 200 mm × 200 mm, as shown in Figure 3c. The internal room temperature was recorded in real-time using a data-logger and thermocouples (Type K, ±0.3 °C), which were placed in the center of the small test room.

In this experiment, a fan heater was used for heating, while cooling was controlled by the air conditioning system programmed to maintain an ambient temperature at 17 °C. The top EGPG panel was heated for a period of 2.5 h and was then cooled down for another 3.5 h.

#### 2.3.4. Temperature Cycling Test

To test the leakage behavior of EG/P, the thermal cycling chamber was used to accelerate the process of phase change. EG/P was enveloped between two filter paper sheets and compressed by 1 kPa static load as mentioned by Aydın et al. [26], and the samples then suffered different thermal cycles from 0 °C to 40 °C with 2 °C/min heating/cooling rate.

In addition, to know the change of mechanical strength after thermal cycling, the flexural and compressive strength of EGPG was evaluated again. After 7 days curing time in an indoor environment, the samples with a size of 40 mm × 40 mm × 160 mm were placed in the thermal cycling chamber for 500 thermal cycles from 10 °C to 40 °C with a 2 °C/min heating/cooling rate.

#### 2.3.5. Strength Tests

After 7 days curing time and 500 thermal cycles (from 10 °C to 40 °C), the 40 mm × 40 mm × 160 mm samples were tested according to the requirements of the China national standard of GB/T 176167.3-1999 (test methods of strength of gypsum). Mechanical loading rates for flexural strength and compressive strength tests were 50 ± 10 N/s and 2.4 ± 0.2 KN/s, respectively.

#### 2.3.6. Chemical Structure Tests

X-ray diffraction (XRD, Bruker D8 Advance, Karlsruhe, Germany) was using an angle range from 5° to 80° was used to measure any change in the material phase of the gypsum after thermal cycling.

## 3. Results and Discussion

### 3.1 Characterization of EG/P

#### 3.1.1. The Microstructure of EG and EG/P

**Figure 4:**
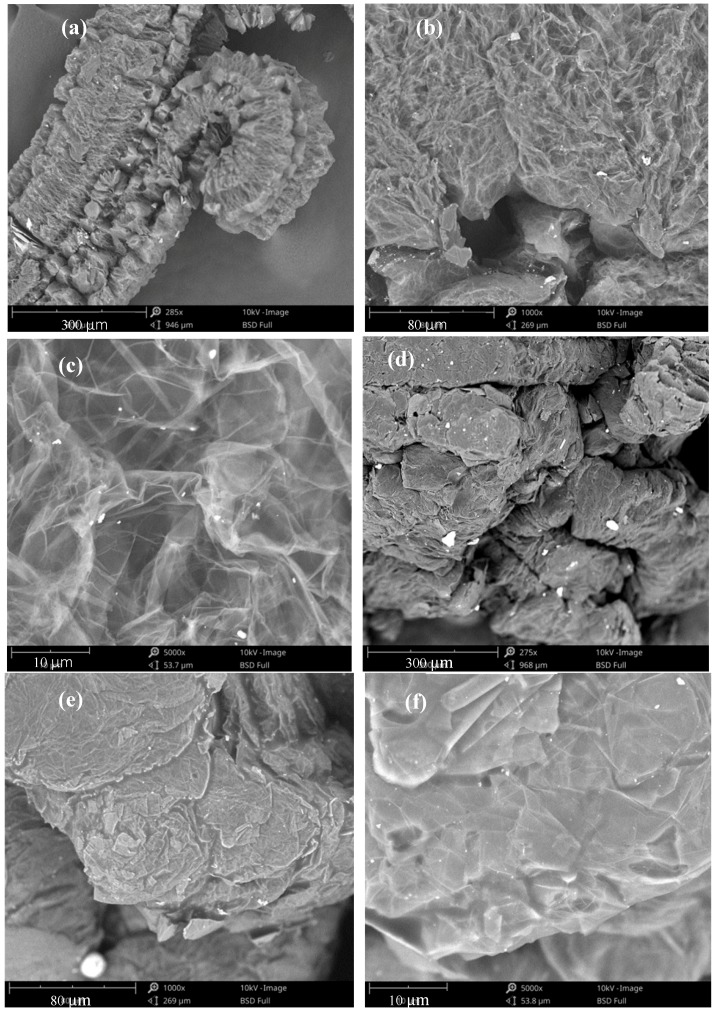
SEM images of EG and EG/P: (**a**) 285× magnification of EG; (**b**) 1000× magnification of EG; (**c**) 5000× magnification of EG; (**d**) 280× magnification of EG/P; (**e**) 1000× magnification of EG/P; (**f**) 5000× magnification of EG/P.

Figure 4 shows the SEM images of EG and EG/P at different magnifications. The pores observed in the EG can be classified into one of three categories: (1) Interconnected and worm-like macrospores; (2) fractured pores on the surface; and (3) internal mesopores. Figure 4a shows a worm-like shape of EG and the pores on the surface are seen to be loose under a magnification of 285×. At a magnification of 1000 times, a large number of pores can be seen on the surface, as in Figure 4b. The network of mesopores in the EG are made by transparent and lamellar graphite, and can be observed at 5000 times magnification, as shown in Figure 4c. These large volume mesopores are able to absorb a large quantity of paraffin, which does not leak when in the liquid phase because of capillary action and surface tension [9,27].

Figure 4d shows the microstructure of EG/P magnified 280 times. From Figure 4d, it can be seen that the surface of EG is more compacted and the EG is stuck together after it absorbs paraffin. This may be due to residual paraffin on the surface of the EG, which acts as a binder to stick adjacent EG pieces together. Higher resolution images of EG are shown in Figure 4e,f, with a magnification of 1000× and 5000×. Compared with Figure 4b,c, the larger scale mesopores disappear and the denser microstructure is observed, indicating that the interior pores play a primary role on the absorption capacity. These results were consistent with Ren et al. [28], who presented that the mesopore sizes of EG is typically in the range of 3–5 nm, which could generate high capillary force and tension force. Thus, the PCM tends to adhere to the internal mesopore of EG. Additionally, they also demonstrated that there was little change on the enthalpy of EG/Ca(NO_3_)_2_-NaNO_3_ after 500 thermal cycles, indicating it has good thermal reliability for a long term period. It can be deducted that the leakage of paraffin from the interior of EG would not occur.

#### 3.1.2. The Thermal Properties of EG/P

**Figure 5:**
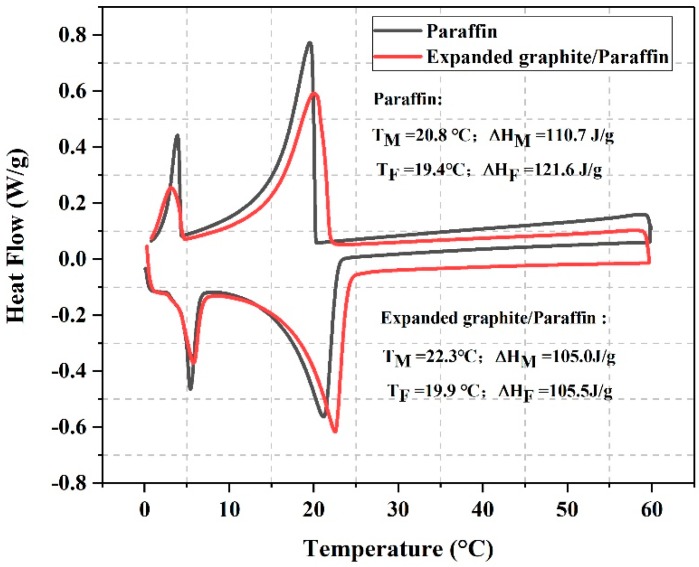
DSC (Differential Scanning Calorimeter) curves of paraffin and expanded graphite/paraffin.

The thermo-physical properties of paraffin and EG/P were determined by DSC. As can be seen from Figure 5, the onset melting temperature of paraffin and EG/P are 20.8 °C and 22.3 °C, while its latent heat values are 110.7 J/g and 105.0 J/g, respectively. It can also see that the EG/P had a relatively higher onset melting temperature than that of pure paraffin. That may be explained by the Clapeyron-Clausius equation [29]:

(1) $$\ln\frac{T2}{T1}=\frac{\Delta_{\alpha }^{\beta }V_{m}}{\Delta_{\alpha }^{\beta }H_{m}}\left(P2-P1\right)$$

where, *T*1 and *T*2 represent the phase change temperature; $\Delta_{\alpha }^{\beta }V_{m}$ and $\Delta_{\alpha }^{\beta }H_{m}$ are the volume change and enthalpy change during the phase change from α-phase (solid state) to β-phase (liquid state); and *P*1 and *P*2 are the ambient pressures during the phase change. When the paraffin is melted, the enthalpy ($\Delta_{s}^{l}H_{m}$) and volume ($\Delta_{s}^{l}V_{m}$) of the paraffin would increase and pressure (*P*2 > *P*1) also increases [30], thus leading to an increase of the melting temperature.

With respect to the cooling process, it can be found from Figure 5 that the enthalpy of paraffin and EG/P are 121.6 J/g and 105.5 J/g, respectively. Meanwhile, the onset freezing temperature of EG/P shifted upward to 19.9 °C compared with that of pure paraffin by 19.4 °C. This result can be explained by the crystallization-promoting effect that the network of inner EG performed as a heterogeneous nucleation center to promote the crystallization reaction of paraffin during the crystallization process, resulting in the increase of onset crystallization [31].

In addition, according to the calculation method of Bao et al. [32], the encapsulation efficiency of EG was measured to be 90.6% by comparing the enthalpy of paraffin (116.2 J/g) and EG/P (105.3 J/g). In comparison to the works of Ramakrishnan et al. and Wen et al. [33,34], a higher value of latent heat of EG/P was obtained from this research. In research conducted by Ramakrishnan et al. [33], they prepared a form-stable phase change materials expanded perlite/paraffin and found that its enthalpy just had 80.1 J/g. Wen et al. [34] used diatomite as carrier and capric-lauric acid as PCM to fabricate the diatomite/capric-lauric acid. Although the enthalpy of capric-lauric acid was 141.5 J/g, diatomite/capric-lauric acid only owned 87.3 J/g. Therefore, it could be summarised that the high value of enthalpy achieved here demonstrates the potential of EG/P materials in building energy applications.

**Figure 6:**
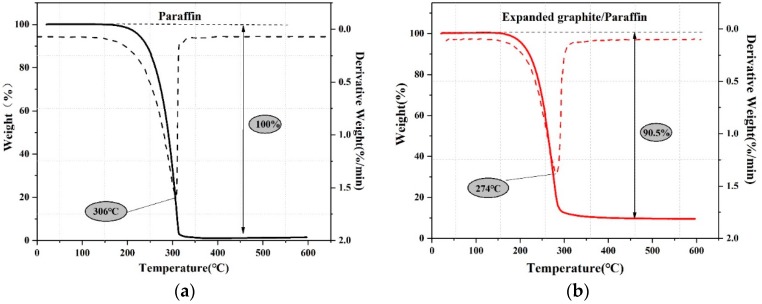
TGA (Thermal Analysis System) and DTG (Derivative Thermogravimetric Analysis) curves of (**a**) paraffin and (**b**) expanded graphite/paraffin.

The thermal decomposition behavior of paraffin and EG/P were evaluated using TGA analysis. As shown in Figure 6, the weight-loss ratio of EG/P is approximately 90.5%, which is roughly equivalent to the mass ratio of paraffin calculated from DSC. This indirectly proves the homogeneity of the samples and the accuracy of the measurement techniques.

It can be seen that both paraffin and EG/P started to decompose at approximately 180 °C. That implies the thermal stability of paraffin is not changed despite being incorporated into EG, and the decomposition temperature was suitable for the engineering application. However, a significant difference between paraffin and EG/P can also be found in Figure 6. When the EG/P is heated to 274 °C, the paraffin inside the EG is completely decomposed, while pure paraffin must be heated to 306 °C. A similar result can be found by the research of Zhang et al. [31]. In their study, it can be found that polyethylene glycol started to decompose at 409 °C, while the decomposition temperature of EG/polyethylene glycol occurred at 391 °C. This phenomenon can be explained by the mechanism that EG could provide an enhancement in the thermal conductivity of the material, resulting in a faster response to applied heat and a higher heat transfer efficiency. Based on the above discussion, EP/G, which has a suitable phase change temperature, high enthalpy, and good thermal stability, can be used as an energy storage material for buildings.

#### 3.1.3. The Thermal Reliability of EG/P

**Figure 7:**
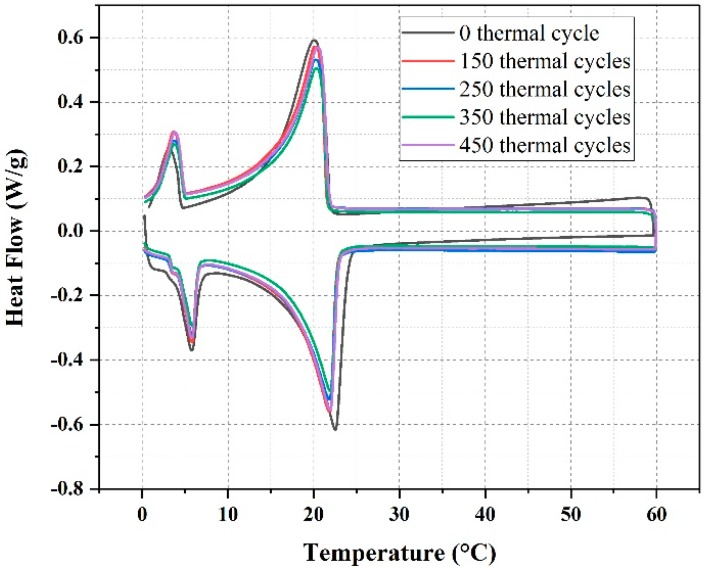
DSC curves of expanded graphite/paraffin under different thermal cycles.

The thermal reliability of EG/P was evaluated using DSC analysis by comparing before and after thermal cycles. As shown in Figure 7 and Table 2, the average enthalpy of EG/P with 150, 250, 350, and 450 thermal cycles are 105.1 J/g, 103.9 J/g, 103.2 J/g, and 103.0 J/g accordingly, which are roughly equivalent to that enthalpy without any thermal cycles. Although the enthalpy of EG/P has a few decreases under thermal cycles, these results can still confirm that the enthalpy of EG/P is guaranteed. Besides, from Table 2, it is clear that the onset melting temperature drops and onset freezing temperature rises after thermal cycles, but are not significant, indicating EG/P has stable melting and freezing points. Therefore, it can be concluded that the newly developed EG/P has excellent thermal reliability.

#### 3.1.4. The Chemical Structure of EG/P

**Figure 8:**
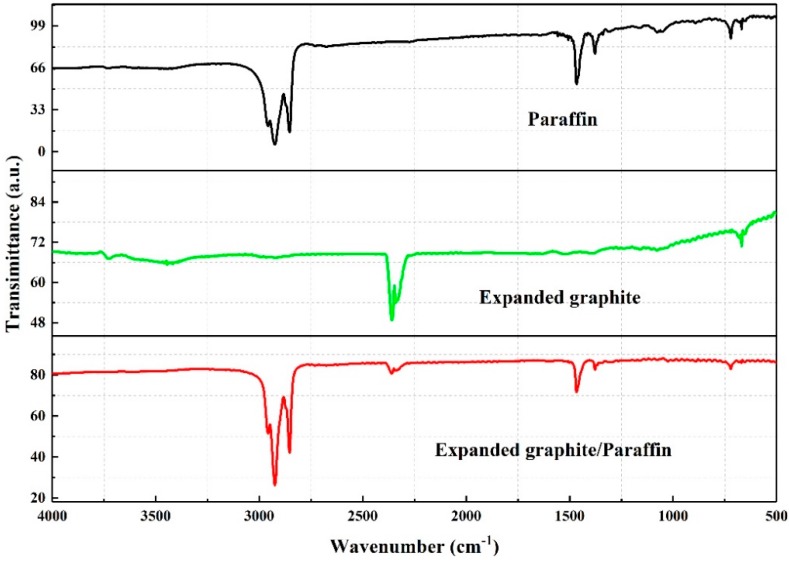
FT-IR spectrum of paraffin, EG, and expanded graphite/paraffin.

The FT-IR spectrum of paraffin, EG, and EG/P between 4000 cm^−1^ to 500 cm^−1^ are shown in Figure 8. The characteristic peak at 719 cm^−1^ that is observed for paraffin, EG, and EG/P is related to the rocking vibration of CH_2_ [35]. The peak observed at 1380 cm^−1^ for EG and EG/P samples is related to the symmetric deformation of CH_3_, and the absorption peak at 1473 cm^−1^ is caused by the asymmetric stretching vibration of the CH_2_ group [36]. A further two strong absorption peaks (2350 cm^−1^ and 2360 cm^−1^) are observed in the EG and EG/P samples, which is due to the stretching vibration of the C–C skeleton in EG [37]. Figure 8 also shows oscillating bands at 2849 cm^−1^ and 2918 cm^−1^ that are linked to the stretching vibrations of CH_2_ and CH_3_, respectively [36]. However, there are no new characteristic absorption bands in the EG/P sample, indicating that the absorption process of paraffin into EG is a physical interaction only with no chemical reaction.

### 3.2. Thermal Conductivity of EGPG

As shown in Table 3, it can be seen that the thermal conductivity of GC increases with an increasing concentration of EG/P. Compared with GC, when 10 wt % and 20 wt % of EG/P are added into GC, the thermal conductivity of GC-10%EG/P and GC-20%EG/P increases by 33.7% and 53.2%, respectively. This improvement was attributed to the high thermal conductivity of EG/P (about 5 W/m·K) [35], and it could improve the thermal conductivity of EGPG. In addition, incorporating CF into the GC also improved thermal conductivity. The thermal conductivity of GC-10%EG/P and GC-20%EG/P was increased by 36.0% and 28.6%, respectively, with the addition of 1 wt % of CF. It can also be expected that the thermal exchange rate of GC-10%EG/P and GC-20%EG/P will also improve significantly with the inclusion of CF because of the high thermal conductivity that is achieved.

### 3.3. The Thermo-Regulated Performance of EGPG

According to Equation (2), the energy storage capacity of EGPG can be calculated, and Table 3 shows the results for each gypsum board.

(2) $$\Delta H_{EGPG}=\frac{\Delta H_{EG/P}\times \;m_{EG/P}}{m_{gypsum}+\;m_{water}+m_{EG/P}+m_{CF}}$$

where Δ*H_EGPG_* and Δ*H_EG_*_/*P*_ represent the energy storage capacity of EGPG and EG/P, respectively; and *m_EG_*_/*P*_, *m_gypsum_*, *m_water_*, and *m_CF_* present the weight of EG/P, gypsum, water, and CF accordingly.

**Figure 9:**
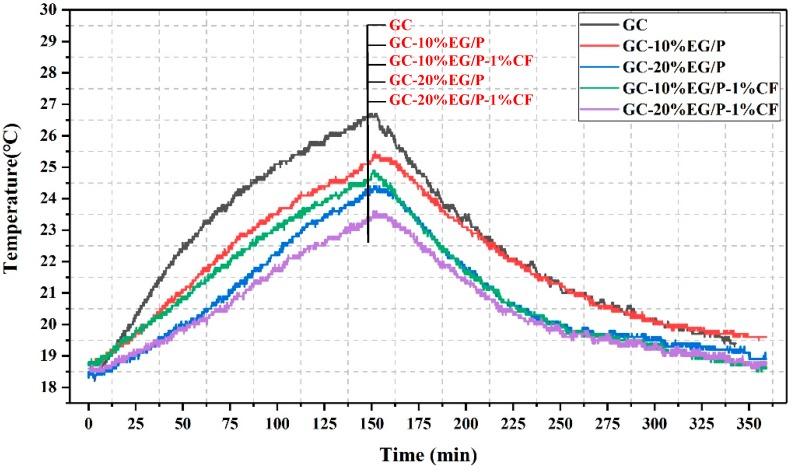
Temperature curves of center point of five small house models.

The thermo-regulated performance of EGPG is evaluated through a self-designed room model. As seen in Figure 9, a similar trend of the temperature variation with time is observed for all EGPG groups. It also can observed that GC has a faster heating and cooling process compared with the other groups. This implies that the GC group had a poor energy storage capacity. However, the test panels containing EG/P show improved temperature regulation properties when compared to GC. That is due to the inclusion of EG/P, which has a high energy storage capacity and is able to absorb a significant amount of heat during the heating phase (0 to 150 min). The heat stored by the EG/P is then released to slow the rate of decreasing temperatures during the cooling phase. Moreover, the thermo-regulated performance of the EGPG was even more effective with an increase in EG/P content. For example, when the highest measured temperature of GC is reached at 26.7 °C (maximum temperature, 159 min), the temperature of GC-10%EG/P and GC-20%EG/P were measured to be 25.4 °C and 24.3 °C, respectively. This represents an improvement compared with the reference GC for a GC-10%EG/P and GC-20%EG/P of 1.3 °C and 2.4 °C, respectively. According to the research of Mourid et al. [38], a PCM coating of 5.3 mm thickness on a standard wall could lead to a temperature increase of about 6 °C during the night and reduce energy consumption by 20% compared to the reference case. In addition to reducing the energy consumption, our previous research [39] shows that a 4 mm thick PCM panel integrated into a wall could improve the energy efficiency rate by 65.3%, and it is equivalent to reducing CO_2_ greenhouse gases emissions by 65.28%. This indicates that EGPG materials are able to reduce temperature fluctuations, resulting in reduced electricity consumption of heating and cooling systems, which has the effect of improving the power generation management and reducing environmental pollution to achieve an improved economic outcome.

The effect of adding CF to EGPG can also be seen in Figure 9. GC-10%EG/P-1%CF and GC-20%EG/P-1%CF show an improved thermo-regulated performance than that of GC-10%EG/P and GC-20%EG/P. Based on the results of Section 3.2, it can be deduced that CF increases the thermal conductivity of GC-10%EG/P and GC-20%EG/P, and thus improves the heat transfer rate of EG/P within the EGPG matrix. Specifically, the maximum temperature of GC-10%EG/P-1%CF and GC-20%EG/P-1%CF decreased by 0.6 °C and 0.8 °C compared with GC-10%EG/P and GC-20%EG/P, respectively. Hence, it can be concluded that the matrix with the highest thermal conductivity provides the greatest increase in the heat transfer rate to the PCM, thus improving the thermo-regulated performance of structural-functional integrated building materials.

### 3.4. The Mechanical Properties of EGPG

**Figure 10:**
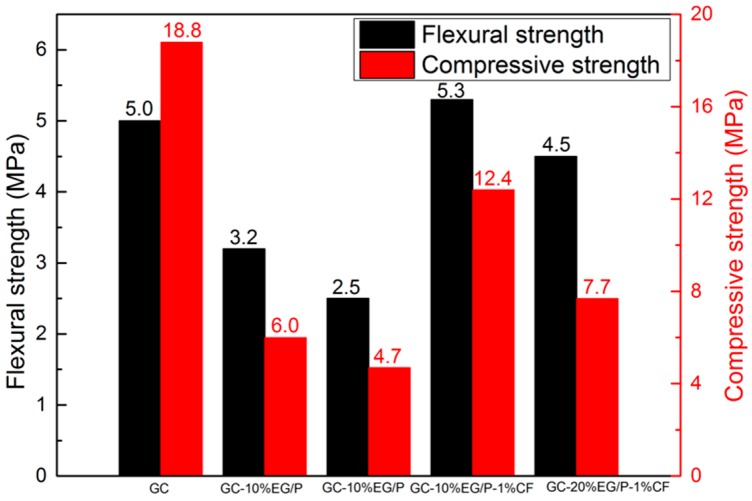
The mechanical strength of EGPG after seven days curing time.

The mechanical properties of EGPG were tested after seven days curing time under ambient conditions, and the results are shown in Figure 10. It was found that with an increasing EG/P content, a decreasing trend in the flexural and compressive strength was observed. For example, when 10 wt % and 20 wt % of EG/P were mixed with GC, the flexural strength of GC-10% EG/P and GC-20% EG/P was reduced by 36.0% and 50.0% compared with the reference GC material, respectively. The compressive strength of GC-10%EG/P and GC-20%EG/P was found to decrease by 61.2% and 72.8%. This result may be due to the fact that a higher total porosity can be generated due to the addition of EG/P, which leads to the observed reduction in mechanical strength [40]. In the research conducted by Pilehvar et al. [41], the compressive strength of cement had a large reduction from 60 MPa to 35 MPa at 28 days when 20% microencapsulted PCM was incorporated. They explained that this pheromone is because the lower stiffness and strength of EG/P could weaken the mechanical strength of GC. However, 1 wt % CF has been found to improve the mechanical strength of EGPG. The flexural strength of GC-10%EG/P-1%CF is 12.4 MPa, which is 51.6% higher than that of GC-10%EG/P, while no change in compressive strength was observed. These results were consistent with Zhu et al. [42], who reported that the fibers with high bond strength increase the brittleness of plaster gypsum-based materials. In their study, the flexural strength of gypsum increased by 91.5% from 2.1 MPa to 4.04 MPa as the addition of 1.2% polyvinyl alcohol fiber with the length of 12 mm.

**Figure 11:**
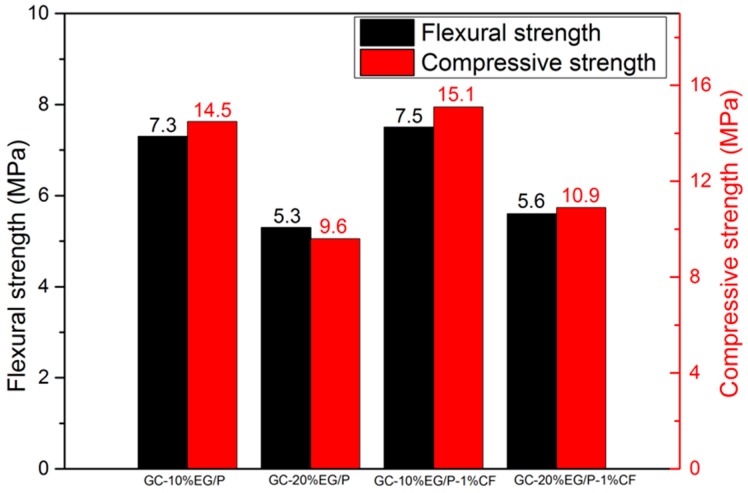
The mechanical strength of EGPG after thermal cycling.

**Figure 12:**
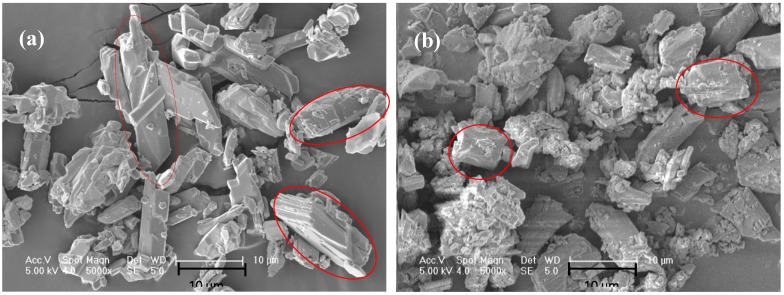
The morphology of gypsum: (**a**) Before thermal cycling; (**b**) after thermal cycling.

**Figure 13:**
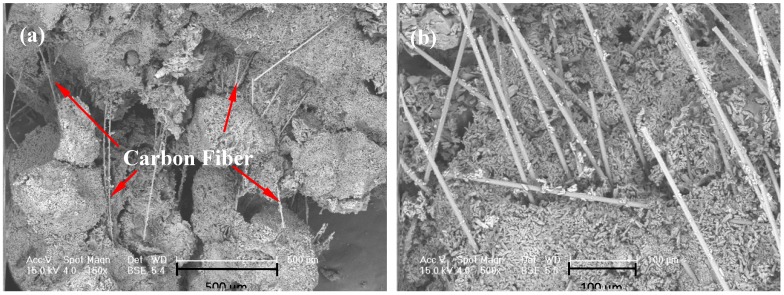
The microstructure of EGPG with CF (Carbon Fiber): (**a**) CF in the cracks of EGPG; (**b**) the surface of EGPG with CF after fracture.

**Figure 14:**
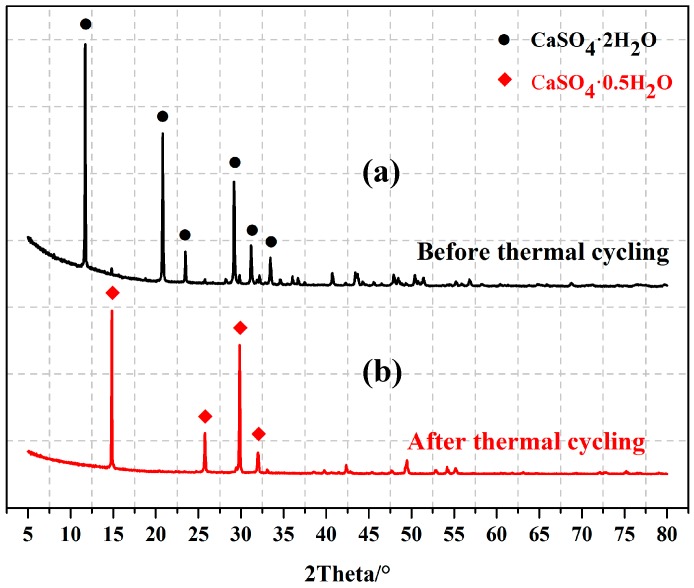
The XRD pattern of gypsum: (**a**) Before thermal cycling; (**b**) after thermal cycling.

A temperature cycling test was used to measure the change in mechanical strength of EGPG materials under service conditions. Figure 11 shows the results of the mechanical strength after 500 heating/cooling cycles from 10 to 40 °C. It is seen that the flexural and compressive strengths of EGPG are doubled after temperature cycling compared with samples after seven days curing at room temperature. Based on the results of SEM and XRD, dehydration of the gypsum is identified as the main cause of the higher mechanical strength. The morphology of gypsum before temperature cycling, as shown in Figure 12a, is calcium sulfate dihydrate (CaSO_4_·2H_2_O), which has rod and thick plate-like crystals measuring 6–15 μm. In comparison, irregular block crystals (calcium sulfate hemihydrate-CaSO_4_·0.5H_2_O, 2–10 μm) are observed after thermal cycling as shown in Figure 12b, which has a positive effect on improving the mechanical strength. Figure 13 shows that CF distributes in the cracks and on the surface of EGPG, which contributes to the higher mechanical strength. Besides, XRD results also have confirmed that CaSO_4_·2H_2_O transformed to CaSO_4_·0.5H_2_O after thermal cycling, as shown in Figure 14. As shown from the sample before thermal cycling (black curve), the characteristic peaks are mainly generated at 11.7°, 20.6°, 23.6°, 29.2°, 31.1°, and 33.4°, which belong to CaSO_4_·2H_2_O [43]. However, in the sample after thermal cycling (red curve), the characteristic peaks are identified at 14.9°, 25.8°, 29.7°, and 32.0°, corresponding to the peak of CaSO_4_·0.5H_2_O.

## 4. Conclusions

In this study, EG/P was developed for use in a gypsum-based thermal energy storage material for application as interior partitions or building envelope materials. Carbon fiber (CF) was used to improve the thermal conductivity and mechanical strength of the gypsum matrix to increase the rate of heat exchange of EG/P and allow its use in a wide range of building applications. The following conclusions can be drawn from the experimental findings:

1. EG/P was prepared successfully by the vacuum impregnation method, which produces material with a latent heat storage capacity of 105.3 J/g and phase change temperature of 22.28 °C. The results of FT-IR reveal that the components within EG/P are chemically compatible, and TGA proves that EG/P can meet the requirements for thermal stability. As a result, EG/P is demonstrated as a potential candidate for buildings as part of overall efficient energy management scheme.
2. Both EG/P and CF improved the thermal conductivity of gypsum (GC). Compared with GC, the thermal conductivity of the GC-10%EG/P and GC-20%EG/P was improved by 33.7% and 53.2%, respectively. When 1 wt % CF was added, the thermal conductivity of GC-10%EG/P and GC-20%EG/P further increases by 36.0% and 28.6%, respectively.
3. EG/P can significantly reduce temperature fluctuations. The maximum temperature of indoor center position decreased by 2.4 °C with the addition of 20 wt % EG/P into GC. CF also has a role in increasing the rate of heat transfer to EG/P within gypsum, and thus the greatest improvement in temperature was 3.2 °C, observed between GC-20%EG/P-1%CF and GC.
4. With the increase of EG/P content, the flexural and compressive strengths of EGPG demonstrate a dramatically decreasing trend. However, the presence of CF can improve the flexural strength of GC-20%EG/P by 51.6%, with no change in compressive strength.
5. After 500 heating/cooling cycles, the mechanical properties of EGPG increased by about 100%, which is attributed to the dehydration of calcium sulfate dihydrate.

## Figures and Tables

**Table 1:** The mix proportions of EGPG (expanded graphite/paraffin gypsum-based composite materials).

No.	Gypsum (g)	Water (g)	EG/P (g)	CF (g)
GC	3600	1300	--	--
GC-10%EG/P	3600	1300	360	--
GC-20%EG/P	3600	1300	720	--
GC-10%EG/P-1%CF	3600	1300	360	36
GC-20%EG/P-1%CF	3600	1300	720	36

Note: GC represents the gypsum control group; GC-10%EG/P represents gypsum mixed with 10%EG/P; GC-10%EG/P-1%CF represents gypsum mixed with 10%EG/P and 1%CF.

**Table 2:** The thermal properties of expanded graphite/paraffin under different thermal cycles.

Cycles	Melting Temperature (°C)	Melting Enthalpy (J/g)	Freezing Temperature (°C)	Freezing Enthalpy (J/g)
0	22.3	105.0	19.9	105.5
150	22.0	104.9	20.2	105.2
250	22.0	103.3	20.2	104.5
350	22.1	102.7	20.3	103.8
450	22.1	101.8	20.3	104.1

**Table 3:** The thermal conductivity of EGPG.

No.	Thermal Conductivity (W/m·K)	Energy Storage Capacity (J/g)
GC	0.742	0
GC-10%EG/P	0.992	7.25
GC-20%EG/P	1.137	13.65
GC-10%EG/P-1%CF	1.350	7.20
GC-20%EG/P-1%CF	1.462	13.57

## Abbreviations

PCMs	Phase Change Materials
EG	Expanded graphite
EG/P	Expanded graphite/Paraffin composites
SSPCM	Stabilized-shape Phase Change Material
EGPG	Expanded graphite/paraffin gypsum-based composite material
CF	Carbon Fiber
